# Flecainide How and When: A Practical Guide in Supraventricular Arrhythmias

**DOI:** 10.3390/jcm10071456

**Published:** 2021-04-02

**Authors:** Carlo Lavalle, Michele Magnocavallo, Martina Straito, Luca Santini, Giovanni Battista Forleo, Massimo Grimaldi, Roberto Badagliacca, Luigi Lanata, Renato Pietro Ricci

**Affiliations:** 1 Department of Cardiovascular, Respiratory, Nephrological, Anesthesiological and Geriatric Sciences, “Sapienza” University of Rome, Policlinico Umberto I, 00161 Rome, Italy; michelefg91@gmail.com (M.M.); martina.straito@uniroma1.it (M.S.); roberto.badagliacca@uniroma1.it (R.B.); 2 Department of Cardiology, Ospedale GB Grassi, 00121 Ostia, Italy; lucasantinimd@gmail.com; 3 Department of Cardiology, Azienda Ospedaliera-Universitaria “Luigi Sacco”, 20057 Milan, Italy; forleo@me.com; 4 Department of Cardiology, Ospedale Generale Regionale F. Miulli, Acquaviva delle Fonti, 70021 Bari, Italy; fiatric@hotmail.com; 5 Medical Affairs Department, Dompé Farmaceutici SpA, 20057 Milan, Italy; Luigi.Lanata@dompe.com; 6 CardioArrhythmology Center, 00161 Rome, Italy; renatopietroricci@gmail.com

**Keywords:** flecainide, flecainide controlled-release, atrial fibrillation, CAST, supraventricular arrhythmias

## Abstract

Transcatheter ablation was increasingly and successfully used to treat symptomatic drug refractory patients affected by supraventricular arrhythmias. Antiarrhythmic drug treatment still plays a major role in patient management, alone or combined with non-pharmacological therapies. Flecainide is an IC antiarrhythmic drug approved in 1984 from the Food and Drug Administration for the suppression of sustained ventricular tachycardia and later for acute cardioversion of atrial fibrillation and for sinus rhythm maintenance. Currently, flecainide is mostly used for sinus rhythm maintenance in atrial fibrillation (AF) patients without structural cardiomyopathy although recent studies enrolling different patient populations have demonstrated a good effectiveness and safety profile. How should we interpret the results of the CAST after the latest evidence? Is it possible to expand the indications of flecainide, and therefore, its use? This review aims to highlight the main characteristics of flecainide, as well as its optimal clinical use, delineating drug indications and contraindications and appropriate monitoring, based on the most recent evidence.

## 1. Introduction

In the recent years several non-pharmacological therapies, in particular transcatheter ablation, have been increasingly and successfully used to treat symptomatic drug refractory patients affected by supraventricular arrhythmias (SVT), especially atrial fibrillation (AF) [1,2]. Nevertheless, antiarrhythmic drug treatment still plays a major role in patient management, alone or combined with non-pharmacological therapies.

Flecainide is an IC antiarrhythmic drug approved in 1984 from the Food and Drug Administration for the suppression of sustained ventricular tachycardia (VT) and later for AF acute cardioversion and for sinus rhythm maintenance.

Currently, flecainide is mostly administered for sinus rhythm maintenance and, having regard to its effectiveness and safety profile, it may be considered underused [3,4,5]. The CAST study published in 1991 has strongly conditioned and limited the actual use of flecainide in clinical practice. The study was prematurely dismissed due to excess of mortality among patients treated with IC agents with a significant greater number of death and cardiac arrest due to arrhythmia than patients treated with placebo [6]. It is necessary to underline that many patients who died during the study had depressed left ventricular ejection fraction and intraventricular (IV) impulse disturbance, two conditions currently contraindicating the use of flecainide.

Differently from the CAST results, recent studies, enrolling different patient populations, have demonstrated a good safety profile of the drug combined with good clinical efficacy. This review aims to highlight the main characteristics of flecainide, as well as its optimal clinical use, delineating drug indications and contraindications and appropriate monitoring, based on the most recent evidence.

## 2. Flecainide Pharmacology

### 2.1. Pharmacokinetics

Flecainide is nearly completely absorbed from the gastrointestinal tract (bioavailability: 85–90%) [7] reaching serum concentration peak in 1–3 h. Concentration from 0.2 to 1 mcg/mL provide the greatest therapeutic benefit whereas value higher than 0.7 to 1 mcg/mL have been associated with increased adverse effects [8]. The apparent volume of distribution is wide, about 40% of drug was binded to plasma proteins [9]. Flecainide is metabolized in the liver via cytochrome (CYP2D6 and CYP1A2), and then excreted in the urine. About 30% of an orally administered dose escapes liver metabolism and is excreted in the urine unchanged [7]. The half-life is about 20 h (range: 12–27 h) and it may be prolonged until 70 h in patients with heart failure, renal disease (creatinine clearance < 50 mL/min) and liver disease [10].

### 2.2. Pharmacodynamics

Flecainide works blocking the open-state fast inward Na^+^ channel Nav 1.5 in a rate- and voltage-dependent manner, reducing the rise of phase 0 of the action potential especially in His-Purkinje tissue and ventricular muscle [10,11,12]. Furthermore, flecainide at low dose inhibits rapid component of the delayed rectifier K^+^ current (I_Kr_) and at higher concentrations inhibits K_ito_ channels [13,14,15]. Overall, flecainide prolongs the duration of action potential and effective refractory period in ventricular fibers while they are both shortened in the Purkinje system, an effect probably consistent with Na^+^ channel blockade [14,15,16]. The drug also inhibits ryanodine receptor 2 reducing calcium sparks and thus arrhythmogenic calcium currents [17]. Moreover, flecainide reduces Na^+^ and Ca^2+^ inflow in myocardial cells and exerts a negative inotropic effect reducing cardiac output and stroke volume, especially for patients with coronary artery disease or left ventricular (LV) disfunction [18,19,20,21,22,23].

### 2.3. Controlled Release Flecainide

Controlled release flecainide allows a once-a-day administration. The pharmacokinetic profile is characterized by a reduced and delayed reaching maximum concentration and lower fluctuations of plasma concentrations during a dosing interval compared with immediate-release form. Serum concentration peak is reached in 26 h, the steady state plasma level is reached after 4–5 days ranging from 0.27 to 0.33 mcg/mL far from plasma level at risk of side effects [24,25]. Controlled release form improves treatment compliance and reduces the risk of side effects and interactions with other drugs preserving clinical benefit.

## 3. What Does “Structural Heart Disease” Mean? A Critical View

Based on the findings of the CAST trial, it reasonable consider how flecainide exerts a proarrhythmic effect in patients with recent acute myocardial infarction (AMI) and/or LV disfunction [6]. CAST patients were eligible for enrollment six days to two years after AMI if they had an average of six or more premature ventricular contraction per hour and a LV function < 55%. Unfortunately, the study was prematurely dismissed due to an increased proarrhythmic risk among patients treated with IC agents than patients treated with placebo [6]. For this reason, the European Society of Cardiology in 2020 Guidelines for the management of AF contraindicated the use of flecainide in patients affected by structural heart disease [1]. Despite this, a critical appraisal of the CAST trial is necessary; firstly, only in 17% of the enrolled patients a complete revascularization was performed, a clinical scenario now less and less frequent [6]. Moreover, a CAST sub analyses showed that during the late post-myocardial infarction period, therapy with flecainide was associated with a steeper increase in death/cardiac arrest rate in the non-Q-AMI group than in the Q-AMI group [26]. Lastly, 48% of CAST patients had severe left ventricular dysfunction. All these patients could have an augmented pro-arrhythmic risk, and therefore, it may be reasonable to perform a more detailed stratification of ischemic heart disease (Is the arrhythmia scar-related? Is there a critical coronary stenosis?).

Practice guidelines extended the findings of CAST to all IC agents and structural heart disease such as congenital heart disease, valvular or significant myocardial heart disease, although evidence are scarce. In recent years, some observational studies have shown promising results on the use of flecainide in patients with non-ischemic structural heart disease.

Flecainide can be useful in long QT syndrome type 3, an arrhythmogenic cardiomyopathy caused by gain-of-function mutations in the SCN5A-encoded Nav1.5 sodium channel involving a pathological increase in late sodium current and, consequently, prolonging QTc [27,28,29]. Long-term flecainide therapy shortened QTc interval and is relatively safe and effective in patients affected by Long QT syndrome type 3; in particular, no cardiac events occurred among patients who were fully compliant with flecainide administration while in patients who discontinued therapy a cardiac event has been observed in 30% of them [30].

Hyman et al., evaluated the safety and efficacy of premature ventricular contraction suppression with Class IC antiarrhythmic drugs in 20 patients with extrasystole-induced cardiomyopathy [31,32]. A satisfactory premature ventricular contraction suppression rate was obtained, and no sustained ventricular arrhythmias or sudden cardiac deaths were reported [31]. Moreover, the addition of flecainide in combination with sotalol or metoprolol demonstrated to be safe and effective in controlling recurrent arrhythmias in patients with arrhythmogenic right ventricular cardiomyopathy [33]. Furthermore, in patients with AF and left ventricular hypertrophic (ventricular wall thickness ≥ 1.4 cm) treatment with class IC agents did not have higher mortality compared with patients treated with amiodarone [34].

**Figure 1:**
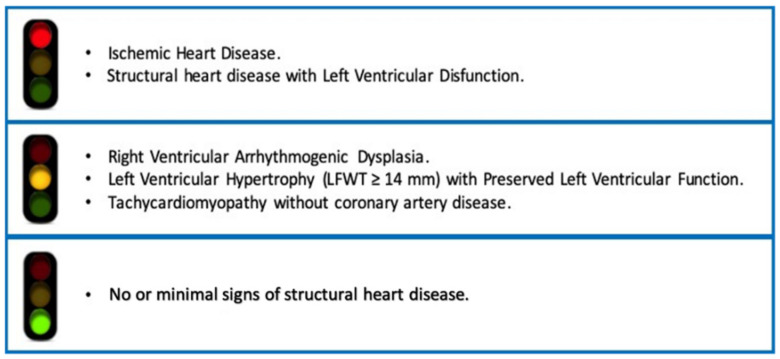
Current evidence on the use of flecainide. Structural heart disease is any abnormality, or defect, of the heart muscle or the heart valves.

Data available are still incomplete and further studies are recommended. In the meanwhile, the criteria listed in the Figure 1 may drive the clinical choice in individual patients.

## 4. Safety Data

Considering the pharmacodynamic effects of flecainide, is not surprising that it prolongs the PR (17–29%), the QT (4–11%) interval and the QRS complex (11–27%) [10]. It must be considered that most of the QT prolongation is due to the widening of the QRS complex, so that the JT interval and the rate-corrected QT interval remain unchanged or slightly increase (3–8%) [35,36]. An important proarrhythmic effect (3–5% of cases) is conversion of AF in atrial flutter with slow atrial rate (flutter IC) that may result in 1:1 atrioventricular (AV) conduction with high ventricular response and large QRS [37,38]. Concomitant therapy with AV blockade (β-blockers, verapamil, diltiazem, digoxin) could avoid this pro-arrhythmic effect [38]. Moreover, QRS duration (>120 ms), advanced kidney failure (creatinine clearance < 30 mL/min/1.73 m^2^), electrolyte abnormalities increase pro-arrhythmic effect of flecainide and should be carefully monitored [38].

Overall, a metanalysis of 122 prospective studies demonstrated that in patients with SVT and no significant LV impairment, proarrhythmic events were significantly lower with flecainide than placebo (2.7% vs. 4.8%; *p* < 0.001) without significant differences in terms of total mortality [39].

Lastly, due to flecainide effect on the Na channels, the major non-cardiac side effects are related to its anesthetic properties; the most frequent are dizziness and visual disturbances while headache, gastrointestinal disturbances and metallic taste are rare [40].

### 4.1. Patients with Pacemaker and Implantable Cardioverter Defibrillator

In the last few years, use of implantable cardiac electronic devices has become increasingly common and at least 50% of these patients may develop AF requiring antiarrhythmic therapy [41]. Early raised issue of negative effects of flecainide on pacing and defibrillation threshold are not a concern anymore due to progress in lead technology, automatic setting of pacemaker output and use of biphasic high energy shocks [42]. On the other hand, antiarrhythmic drugs may enhance rhythm control in patients with pacemaker and AF in a hybrid approach [43]. Boriani demonstrated that use of flecainide was associated with lengthened atrial tachycardia cycles and consequently higher atrial anti-tachycardia pacing efficacies [43]. This effect was probably correlated either to prolongation of atrial wavelength or widening of the temporal excitable gap during AF. Atrial anti-tachycardia pacing cannot terminate AF, but it can terminate atrial tachycardia episodes that are the first step in AF disease history [44,45]. For these reasons, flecainide administration could increase atrial anti-tachycardia pacing efficacy that represented an independent predictor of permanent or persistent AF risk.

Otherwise, in patients with VT and implantable cardioverter defibrillator, flecainide may induce lengthening of cycle length of VT due to IV conduction delay resulting in out-of-window VT which will not be treated by defibrillator. On the contrary, in case of SVT, enlargement of QRS morphology induced by flecainide may result in inappropriate shocks [46].

### 4.2. Patients with Sinus Bradycardia and/or AV-IV Conduction Disturbances

The effect of flecainide on the healthy sinoatrial node is irrelevant: sinus rate, sinoatrial conduction and sinus node recovery times are usually not affected by long-term drug administration [47]. On the other hand, administration of flecainide in patients affected by sinus node dysfunction or atrial conduction disorders depresses sinus activity and increases significantly the corrected sinus node recovery time [48]. Due to dose-dependent prolongation of AV and IV conduction, unless a cardiac stimulator is available for emergency cardiac stimulation, flecainide should not be administered in patients with second degree or superior AV block, right or left bundle branch block [8,10,49].

Interaction between antiarrhythmic drugs and autonomic nervous system is very different; while flecainide exerts a mild vagolytic effect, other anti-arrhythmic drugs such as propafenone or amiodarone have anti-adrenergic effect [42,50]. For this reason, flecainide could be the first drug therapy in the maintenance treatment of SVT in patients with physiological bradycardia.

### 4.3. Flecainide in Association with Other Antiarrhythmic Drugs

In some AF patients, a combined anti-arrhythmic strategy may be necessary to maintain sinus rhythm and reduce symptomatic AF recurrences. Flecainide in combination with amiodarone is interesting, not only because it may be effective when the efficacy of each is inadequate as a single-drug therapy, but also because it may allow a reduction in their respective dosages and side effects [51,52,53].

Overall, beta blockers are the most used antiarrhythmic drug in association with flecainide. Capucci et al. have demonstrated that combination therapy with flecainide and metoprolol significantly reduced recurrences of AF at 1-year follow-up when compared with flecainide alone [54,55]. The association of flecainide with other AV blockers like digoxin is less frequent; close monitoring of serum digoxin concentrations is recommended because it could increase by 15–19% with co-administration of flecainide [56,57]. In the same way, the combination of flecainide with verapamil should be use with caution because of potential additive effects on myocardial contractility and on AV conduction [57].

### 4.4. Flecainide in Pregnancy and in Pediatric Population

ESC guidelines recommend avoiding any antiarrhythmic drug during the first trimester of pregnancy [58]. Overall, there is no clear evidence of the teratogenic effect of flecainide. Therefore, it could be used for the treatment of fetal arrhythmias [52].

The treatment of SVTs in the pediatric population is more complex: the immaturity of the cardiac structures can lead to the development of arrhythmias which may resolve spontaneously within the first year [59,60]; moreover, due to the different pharmacokinetics and pharmacodynamics characteristics, administration of drugs in children must be exercised in the extreme caution [61,62,63].

## 5. Vademecum for the Management of Flecainide

A 12-lead electrocardiogram is mandatory before starting the therapy; symptomatic bradycardia, second degree or superior AV block, QRS > 120 ms or Brugada syndrome contraindicate the flecainide prescription. It’s reasonable to perform an echocardiogram to evaluate LV function and exercise stress testing in high-risk patients to exclude the possibility of coronary artery disease. It is strongly suggested to test the first dose under medical observation. The minimum effective plasma concentration of flecainide is about 200 ng/mL while optimal range is between 200 ng/mL and 400 ng/mL [64]. This plasma concentration leads to a QRS prolongation of about 10 ms; a prolongation of 40 ms or more is associated to an increased probability of cardiovascular adverse effects [64].

**Figure 2:**
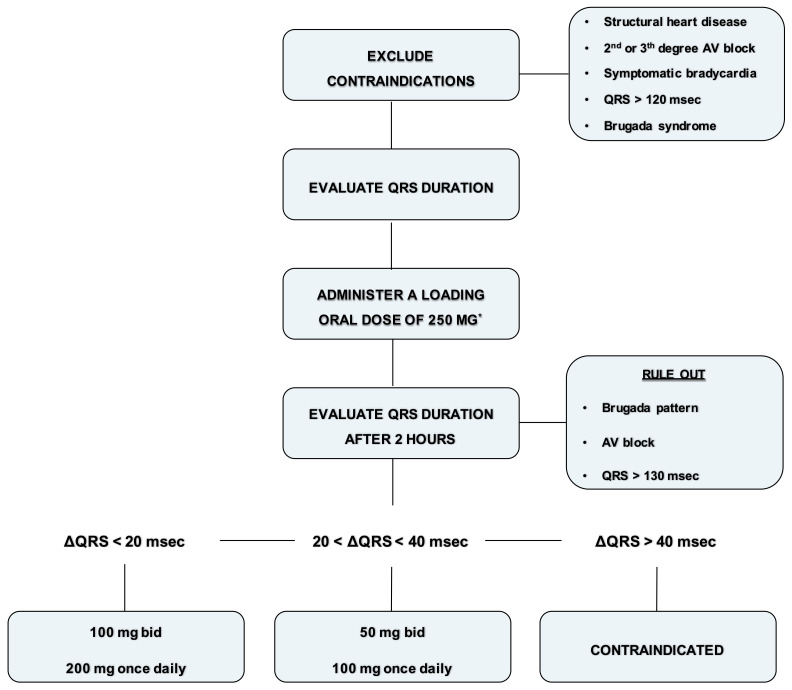
Flowchart for administration of flecainide. * Loading oral dose of 200 mg is recommended if the weight is lower than 70 kg.

A practical approach to flecainide dose ranging, in absence of kidney failure, is as follows (see also—Figure 2):

1. Exclude contraindications (structural heart disease, symptomatic bradycardia, second degree or superior AV block, QRS > 120 ms or Brugada syndrome).
2. Record an ECG with a paper speed of 50 mm/sec and calculate the QRS duration (1 mm = 20 ms).
3. Administer a loading oral dose of 250 mg (200 mg if the weight is lower than 70 kg).
4. At plasma concentration peak, after 90–120 min, evaluate blood pressure and record an ECG with a paper speed of 50 mm/s and calculate the QRS duration.
5. Rule out Brugada ECG pattern and AV block.
  - If the QRS duration is increased within 20 ms, prescribe 100 mg twice daily or 200 mg once daily. Check again the ECG after one week.
  - If the QRS duration is increased between 20 and 40 ms or is wider than 120 ms, prescribe 50 mg twice daily or 100 mg once daily. Check again the ECG after 5 days.
  - If the QRS duration is increased more than 40 ms or is wider than 130 ms, or a Brugada pattern is detected, consider flecainide contraindicated in that patient.

The proarrhythmic risk and other serious adverse effects can be minimized by keeping strict adherence to treatment, limiting the number of drugs prescribed, starting the treatment at low doses that will be increased on the basis of the patient’s response and comorbidities. The recommended dose of flecainide in SVTs are reported in Table 1.

ECG monitoring is suggested in case of drug adjustments or concomitant therapy with other antiarrhythmic drugs, particularly in the elderly and in patients with hepatic and/or renal dysfunction. For appropriately selected patients who have received an initial oral loading dose under monitored conditions, a “pill in the pocket” strategy could be suggested for patient self-administration at the onset of recurrent AF. A practical guide on the management of adverse events due to flecainide is provided in Table 2.

## 6. Flecainide in Atrial Fibrillation

### 6.1. Flecainide in Converting Recent Onset of Atrial Fibrillation

In the acute setting, flecainide is very effective in restoring sinus rhythm with high percentages of success, greater than both propafenone and amiodarone as well as with shorter cardioversion times [65,66,67,68,69,70,71,72,73] (Table 3).

Flecainide is also effective when administered orally; an Italian multicenter study evaluated the efficacy and safety of “pill in the pocket” therapy with flecainide or propafenone in 210 of 268 hospitalized patients for AF onset by less than 48 h who were effectively treated in hospital setting [74]. The pill in the pocket strategy was effective in 94% of patients with an average resolution time of 113 min; adverse events were 7% and in only one case was a high ventricular response atrial flutter. The “pill in the pocket” strategy is currently indicated as a therapeutic strategy in selected patients, with recent onset of AF without significant structural or ischemic heart disease or pro-arrhythmic conditions as Brugada syndrome [1,75,76] and able to self-diagnose AF in which could be avoided the emergency room admission [77,78,79].

### 6.2. Pre-Treatment with Flecainide in Patients Undergoing Electrical Cardioversion

There are strong evidence supporting the use of flecainide prior cardioversion: in a prospective, randomized, double-blinded study were enrolled 54 patients with persistent AF scheduled to electrical cardioversion. Patients that received flecainide before cardioversion had a more successful first shock in comparison to placebo (65% vs. 30% respectively, *p* = 0.04) [80]. Moreover, Boriani et al. demonstrated that flecainide reduces atrial defibrillation threshold increasing procedure success rate in patients with persistent AF [81]. Overall, a pre-treatment with flecainide should be considered in patients at high risk of failure for electrical cardioversion (left atrium dilatation, previous cardioversion failure).

### 6.3. Flecainide in Long Term Rhythm Control

The maintenance of the sinus rhythm is more advantageous than rate-control both in terms of survival and quality of life [82,83,84,85,86,87,88]. According the 2020 ESC Guidelines, catheter ablation is indicated after one failed/intolerant treatment with class I or III drug or to improve symptoms of AF recurrences in patients with paroxysmal and persistent AF [1,89,90]. A rhythm control strategy based on drug administration could be preferable when catheter ablation was hazardous. In this scenario, amiodarone could be considered the most effective in the maintenance of sinus rhythm, but for the high adverse effect rate, a lot of patients withdraw prematurely the treatment [91,92,93]. Otherwise, several studies showed as flecainide was effective in reducing the recurrences of paroxysmal AF and safer when compared to other antiarrhythmic drugs, including amiodarone (Table 4). To reduce adverse event rate a flecainide short-term treatment should be considered for patients with AF who are at increased risk for complications and amiodarone is contraindicated [94]. Indeed, the randomized Flec-SL blinded trial compare flecainide (200–300 mg per day) for four weeks (short-term treatment) with flecainide for six months (long-term treatment) in patients with persistent AF after an effective cardioversion; short-term treatment after cardioversion is less effective than long-term treatment, but in any case, it can prevent most recurrences of AF [92].

## 7. Flecainide in Other Supraventricular Arrhythmias

Although procainamide is the drug of choice in patients with atrio-ventricular reentrant tachycardia, flecainide is effective and safe by directly slowing or blocking conduction over the Na^+^ dependent fast accessory pathway [58]. Flecainide is efficient in approximately 85% to 90% of patients, with 30% reporting an absence of tachycardia [98,99]. Flecainide is also successful in terminating pre-excited AF in hemodynamically stable patients with high-ventricular response and should be considered in the prevention of SVT in pregnant patients with the Wolff–Parkinson–White syndrome [100,101]. However, the gold standard for patients with symptomatic recurrences remains catheter ablation and pharmacological therapy would be reserved for cases where ablation is not desired or feasible [58].

Atrial premature beats and atrial tachycardia are a common finding in older individuals and frequent atrial premature beats are considered a marker of atrial electrical vulnerability and predictors of incident AF [102,103]. The use of flecainide is effective in addition to optimization of medical therapy when catheter ablation was not feasible [104]. Data to support the recommendation for flecainide for maintenance of sinus rhythm in patients with atrial flutter is derived from trials that pooled patients with AF and atrial flutter [105,106]. It is no longer recommended for acute cardioversion of macro-re-entrant atrial arrhythmias and no longer mentioned for chronic therapy of typical atrial flutter [58]. Recommended dose of flecainide in SVTs is reported in Table 1.

## 8. Conclusions

Flecainide is highly effective for the acute termination and for the chronic suppression of AF. An excellent safety profile is described in patients with minimal or no signs of structural heart disease while mounting promising evidence will be available in patients with cardiomyopathy. The “pill in the pocket” approach reduces the need for emergency care and should be more widely employed in patients to achieve rhythm control without long term antiarrhythmic drug exposure and to avoid the necessity for electrical conversion. A 12-lead ECG is required before starting therapy while ECG monitoring is suggested in case of drug adjustments or concomitant therapy with other antiarrhythmic drugs, particularly in the elderly and in patients with hepatic and/or renal dysfunction.

## Figures and Tables

**Table 1:** Recommended dose of flecainide in supraventricular arrhythmias (SVT).

SVT	Recommended Dose	Administration Route
Atrial Fibrillation (Restoration of sinus rhythm)	200–300 mg 2 mg/kg	Oral Intravenous
Atrial Fibrillation (Maintenance of sinus rhythm)	100–200 mg bid 200 mg once daily	Oral
AVNRT/AVRT	50–150 mg bid	Oral
Focal Atrial Tachycardia	50–150 mg bid	Oral

AVNRT: atrioventricular nodal reentrant tachycardia; AVRT: atrioventricular reentrant tachycardia.

**Table 2:** Management of adverse events due to flecainide.

Adverse Event	Incidence	Indication
Drug induced Brugada	<1%	Discontinue
		
QRS increased more than 40 ms or wider than 130 ms	<1%	Discontinue
		
QRS increased more than 20 ms	1–2%	Reduce Dosage
		
Bradyarrhythmia, sinus pause, AV block	1–2%	Discontinue
		
Hypotension	3–5% (mostly with IV)	Reduce Dosage
		
1:1 atrial flutter	3–5%	Discontinue and consider ablate CTI dependent-flutter
		
Worsening heart failure	<1%	Discontinue
		
Extracardiac effects (dizziness, tremor, nausea)	1–2%	Reduce Dosage

AV: atrioventricular; CTI: cavotricuspid isthmus; IV: intravenous.

**Table 3:** Reversion rate of recent-onset atrial fibrillation to sinus rhythm.

Study	Population in Flecainide Arm	AF Duration	Flecainide	Reversion Rate	Adverse Event
Martínez-Marcos et al. [65]	50	≤2 d	Intravenous (2 mg/kg followed by 1 mg/kg at 8 h if not in sinus rhythm)	1 h → 58% 8 h → 82% 12 h → 90%	Transient junctional rhythm: 4% Atrial flutter: 2% Symptomatic hypotension:2% Paresthesia: 4% Total: 12%
Capucci et al. [67]	58	≤7 d	Single oral dose (300 mg)	3 h → 59% 8 h → 78%	Transient junctional rhythm:1.7% Atrial flutter: 3.4%
Crijns et al. [68]	13	≤24 h	Intravenous (2 mg/kg up to 150 mg)	3 h → 77%	-
Boriani et al. [69]	69	≤7 d	Single oral dose (300 mg)	1 h → 13% 3 h → 57% 8 h → 75%	-
Capucci et al. [70]	22	≤7 d	Single oral dose (300 mg)	8 h → 91% 24 h → 95%	no
Romano et al. [71]	138	≤3 d	Intravenous	1 h → 73% 3 h → 80% 6 h → 86% 24 h → 90%	-

**Table 4:** Flecainide for maintenance of sinus rhythm.

Author	*n*. Patient	Type of AF	Compared Treatment	Endpoint of AF Recurrence	Results
Chimienti et al. [82]	200	Paroxysmal	Flecainide vs. Propafenone	Palpitation recurrence on days 15, 30, 90, 180, 270, 360	No difference between flecainide and propafenone
Gulizia et al. [83]	176 with PMK	Paroxysmal	Ic AAD vs. Amiodarone	Time to first occurrence of death, atrial cardioversion, cardiovascular hospitalization or change of AAD	IC AAD non inferior to Amiodarone. Similar AT recurrences
Naccarelli et al. [95]	239	Paroxysmal	Flecainide vs. Quinidine	AF recurrence at 12 months	Flecainide similar efficacy to quinidine but better tolerated
Allot et al. [94]	97	Paroxysmal	Flecainide vs. Propafenone	AF recurrence at 12 months	Flecainide similar efficacy to propafenone
Carunchio et al. [96]	66	Paroxysmal	Flecainide vs. Sotalolo vs. Placebo	AF recurrence at 1, 3, 6 and 12 months	Flecainide similar efficacy to sotalol and superior to placebo
van Wijk et al. [97]	26	Paroxysmal	Flecainide vs. Quinidine	AF recurrence during 3-months follow-up period	Flecainide superior to quinidine

AAD: antiarrhythmic drug, AF: atrial fibrillation; AT: atrial tachycardia; PMK: pacemaker.

## Abbreviations

AF	Atrial Fibrillation
AMI	Acute Myocardial Infarction
AV	AtrioVentricular
IV	IntraVentricular
LV	Left Ventricular
SVT	SupraVentricular Tachycardia
VT	Ventricular Tachycardia
